# Dog bite injuries to humans and the use of breed-specific legislation: a comparison of bites from legislated and non-legislated dog breeds

**DOI:** 10.1186/s13620-017-0101-1

**Published:** 2017-07-21

**Authors:** Nanci Creedon, Páraic S. Ó’Súilleabháin

**Affiliations:** 1 Department of Dog Behaviour, Creedons College, Vicars Road, Cork City, Ireland; 2 School of Psychology, National University of Ireland, Galway, University Road, Galway, Ireland

**Keywords:** Breed-specific legislation, Bite, Public policy, Dog breed, Medical treatment, Bite severity, Dog bite reporting

## Abstract

**Background:**

The primary objective of this study was to investigate if differences in dog bite characteristics exist amongst legislated and non-legislated dog breeds listed under breed-specific legislation in Ireland (age when bitten, anatomical bite locations, triggers for biting, victim’s relationship with the dog, geographical location and owner presence, history of aggression, reporting bite incident to authorities, medical treatment required following the bite, and type of bite inflicted). A second objective of the current study was to investigate dog control officer’s enforcement and perceptions of current legislation. Data for statistical analyses were collated through a nationally advertised survey, with Pearson Chi-square and Fisher’s Exact Test statistical methods employed for analyses. A total of 140 incident surveys were assessed comprising of non-legislated (*n* = 100) and legislated (*n* = 40) dog bite incidents.

**Results:**

Legislated breeds were significantly more likely to be perceived as aggressive and less fearful as triggers for biting compared to non-legislated breeds (*P* = 0.003). Non-legislated breeds were more likely to inflict a bite with the owner present on own property and on a business premises compared to legislated breeds (*P* = 0.036). Non-legislated breeds were more likely to not be reported to the authorities before (*P* = 0.009), and after (*P* = 0.032) the bite occurred compared to legislated breeds. There were no significant differences observed between both groups for; age when the victim was bitten, bite location, relationship with the dog, history of aggression, outcome for the dog, if the dog bit again, and seeing a professional trainer or behaviourist. No significant difference was observed between both legislated and non-legislated groups for medical treatment required following the bite, and the type of bite inflicted.

**Conclusion:**

The present study results did not observe evidence of any differences between legislated and non-legislated for both the medical treatment to victims required following the bite, and the type of bite inflicted. The significant differences in bites being reported to authorities, perceived triggers for biting, and biting locations suggests distinctly differing perceptions relating to risk between legislated and non-legislated dog breeds. Further consequences relating to the introduction of breed-specific legislation in Ireland are discussed.

## Background

In order to minimise potential risks associated with dog bites, governments typically utilise dog breed-specific legislation or non-breed specific legislation. Breed-specific legislation prohibits ownership or places restrictions on dog breeds categorised as ‘dangerous’ or ‘able to inflict greater injuries’ [1]. Non-breed specific legislation includes restrictions targeting irresponsible owners based on exhibited behaviour of their dogs, typically including an educational component [2].

To limit serious dog bite injuries and potential fatalities in Ireland, the Control of Dogs Act 1986 was amended with the inclusion of restrictions on breeds (Control of Dogs Act 1998 Regulations) [3]. The Control of Dogs Act 1998 Regulations places restrictions targeting 11 dog breeds, including dogs that are mixes or possess any strain of listed breeds. The breeds include; American Pit Bull Terrier, Bull Mastiff, Doberman Pinscher, English Bull Terrier, German Shepherd, Japanese Akita, Japanese Tosa, Rhodesian Ridgeback, Rottweiler, Staffordshire Bull Terrier and every dog type known as a Bandog. It was initially thought that the aforementioned breeds (including mixes and strains) possess a greater disposition towards aggression, and as such should be restricted as a public health measure [4]. More recently, it is proposed that legislated dogs have a greater capability of inflicting more severe injuries compared to other non-legislated dogs [5].

Societal attitudes towards dogs are changing in Ireland with increases in dogs being housed in closer proximity to people. While this might suggest a greater threat for dog bites due to this closer proximity, evidence suggests that ‘resident dogs’ who are not fully integrated into family units as being involved in a significant proportion of dog bite fatalities [6]. In other words, dogs who are in close proximity to people form attachments to them, rely on their guidance, and as such account for a lesser rate of dog bite fatalities compared to resident dogs. A further study on dog bites in Ireland found that the breeds most commonly involved in attacks were breeds in the highest numbers within the population [7]. This is supported by further research on dog biting populations which relate to popularity in a geographical location [8]. Recent research has found that dog bite hospitalisations have continued to rise over a 15-year period following the introduction of the current breed-specific legislation in Ireland [1]. The study suggested breed-specific legislation as not being a valid method of reducing incidence rates, and suggested that it may be contributing in part to the rise in dog bites as a result of reinforcing stereotypes of risk pertaining to dog breeds [1].

Research from various other nations have suggested a lack of any efficacy and validity of targeting dog breeds as a dog bite mitigation strategy [8–14]. Conversely, research has observed some reductions in dog bites in a municipality following the enactment of breed-specific legislation [15]. However, once jurisdictions were used as their own controls in a pre/post comparison of incidence of dog-bite hospital admissions, there was no significant reduction in hospitalisations after breed-specific legislation was enacted [15]. A further study reported some reduction in dog bite incidence following the enactment of breed-specific legislation [16]. However, aside from several significant limitations outlined in the study, it is difficult to determine which aspects of the legislation have led to reductions. In other words, the enforcement of accompanying breed-neutral components could have led to some reductions, rather than the actual measures targeting dog breeds. Indeed, employing the statistical methodology of number-needed-to-treat (NNT; commonly used to determine the effectiveness of an intervention) reveals one aspect which makes the targeting of dog breeds at best, impractical. It has been reported that in order to prevent 1 dog-bite hospitalisation in a city or town, in excess of 100,000 dogs of the identified breeds would have to be removed completely from the population [17]. Figures would need to be doubled to prevent a second dog-bite hospitalisation, and so on [17]. Given breed-specific legislation also does not involve complete bans in certain nations (e.g., muzzle restrictions in Ireland), the figures would be considerably higher given the frequency of dog bites in the home when a muzzle is not public policy [17].

Research indicates no fundamental difference in aggression between legislated breeds, and other dog breeds frequently stereotyped as ‘friendly’ [18–20]. However, it remains the case that other group differences between legislated and non-legislated breeds could infer a greater risk of these dog breeds to public health. It is frequently proposed that while legislated breeds may not bite as frequently, in the event of a bite they can inflict greater injury compared to non-legislated breeds of similar size. However, a recent review has investigated claims which have been made in relation to a dog’s bite force ability, and in particular the force sometimes attributed to dog breeds and types frequently legislated for [21]. The review found that research literature have been ‘daisy chaining’ citations which actually do not possess any data, and some not containing any information pertaining to bite force at all [21]. As such, the present study sought to determine if differences exist between legislated and non-legislated dog breeds regarding a host of dog bite characteristics, which included dog bite severity and bite type. In doing so, a primary aim of the present study was to examine various biting characteristics and circumstances attributed to both legislated and non-legislated dog breeds through the collation of survey data from dog bite victims. Given the potential for reinforced stereotypes of aggression and specified behaviour attributed to these breeds, a second aim of the current study was to investigate dog control officer’s perceptions of current legislation which target these dog breeds. Given their central role in the enforcement of legislation targeting specific dog breeds, an examination of their perceptions were crucial to a more complete understanding of the relationship between public health and targeting specific breeds under current legislation.

## Methods

### Sample

The primary study sample constituted a retrospective survey of participants who have ever suffered a dog bite injury within the Republic of Ireland. Surveys were completed and collected between 24 June 2015 and 19 March 2016. The survey was promoted on television, radio, print media, medical centres, and social media throughout Ireland. Any member of the public who had been bitten in the Republic of Ireland, at any stage in their lives, by a dog aged 6 months or older was invited to participate in the survey. The survey data was collated online where all promotional materials relating to the survey directed to. Given legislated breeds do not fall into the small breed category under Kennel Club breed categorisations [22], small breeds were not examined. As previously outlined, this was done in order to increase the validity of comparisons between breeds of similar size. To limit the potential confounding of puppy mouthing, bites from dogs under 6 months were not collated. To control the potential limitation of inaccurate breed identification [23], mixed and unknown dog breeds were not examined. The final sample consisted of 140 dog bite incidents, were categorised as legislated (*n* = 40) and non-legislated (*n* = 100) dog breed bites.

The secondary sample consisted of dog control officers throughout the Republic of Ireland (*N* = 23). Each officer was provided with a web address such that they could anonymously complete their survey. All dog control officers who operate all county and city dog pounds in the Republic of Ireland were contacted. Of the officers contacted, 17 decided to take part with the remaining declaring they did not wish to take part (*n* = 1), did not have the knowledge to respond to the questions (*n* = 1), and repeatedly failing to get the correct officer to take part (*n* = 4). Officers indicated that the following groups operate the dog shelter; Irish Society for Prevention of Cruelty to Animals (ISPCA; *n* = 3), local authority government (*n* = 9), private enterprise (*n* = 4), and not disclosed (*n* = 1).

### Survey sesign

Survey questions included: age when bitten, anatomical location of bite, trigger for the bite, relationship with the dog, owner presence when bite occurred, history of aggressive behaviour, if the dog was reported to the authorities before the bite, if the incident was reported to the authorities after the bite occurred, what was the outcome when reported, (if known) did the dog go on to bite again, and (if known) did the dog owner seek advice from a dog trainer or behaviourist. Relationship with the dog was subdivided into four categories, which included two categories examining if the dog was owned by the victim for either greater than or less than 3 months. This distinction in duration of possession was made based on literature indicating potential duration of time required for a dog to fully acclimatise to their environment [24].

Given the potential for misinterpretation, two measures to determine specific details surrounding the reported bite were employed. Firstly, details surrounding the medical treatment required were of crucial importance. Participants described their injuries in detail in words through an open-ended question, which were later assessed by a certified accident and emergency nurse. These detailed descriptions were then coded by the health care professional into four categories; none (home maintenance); doctor visit, antibiotics and tetanus shot; stitches and regular wound dressing; surgery, fractures and repeat hospital visits. Secondly, the type of bite as indicated by participants within the Dunbar Bite Scale was collated [25]. Both Level 1 bites (no teeth contact) and Level 6 bites (fatality) were not collated. Level 6 bites were not collated given the focus of the present study on examining dog bite incidents from the victim’s perspective. Additionally, there was no availability of information relating to any human fatalities due to dog bite in Ireland. Level 2 bites refers to skin-contact by teeth but no skin-puncture; Level 3 bites refer to a single bite including one to four puncture wounds with no puncture deeper than half the length of the canine’s teeth; Level 4 bites refers to one to four puncture wounds from a single bite with at least one puncture deeper than half the length of the dog teeth; Level 5 bites refer to a multiple-bite incident with at least two Level 4 bites or multiple attack incidents with at least one Level 4 bite in each.

### Data analysis

The statistical package SPSS 22 was used to perform statistical analysis [26]. Data on the variables were organised in cross-tabulations and examined with Pearson’s Chi-square test (*P* ≤ 0.05 chosen as accepted significance level). An a priori computation was conducted to calculate required sample size (1 – β = .80; α = .05; *N* = 44, *w* = .5; *N* = 122, *w* = .3; *N* = 1091, *w* = .1; [27]. Present sample size (*N* = 140) was sufficiently powered to detect medium to large effect sizes. Where the assumption of sample size for cell count was violated in the analysis, Fisher’s Exact Test is reported. Statistical residual outputs were examined to determine locations of any significant effects. Descriptive statistics are displayed in tables to aid discussion. Unknown responses while highlighted descriptively within tables and where relevant were treated as missing data and excluded from the relevant analyses. This approach has been used in existing research [e.g. 10].

## Results

### Breeds, age categories, bite locations and gtriggers for biting

The leading numbers of reported dog bite breeds within the study are illustrated within Table 1. This is descriptive and breed risks cannot be inferred from this as total populations within each category are unknown. An investigation of potential differences between legislated and non-legislated breeds revealed no significant difference with respect to age when bitten (*P* = 0.698; see Table 2). A significant effect for bite location was also not observed (*P* = 0.073). Examination of perceived triggers for biting revealed a significant difference between groups (*P* = 0.003). An investigation of residuals revealed a number of factors contributed to this effect. Firstly, the biting trigger for non-legislated breeds (94.1%) were more likely to be reported as being afraid compared to legislated breeds (5.9%). Secondly, legislated breeds (46.7%) were more likely than expected to be reported as angry as a trigger for biting compared to non-legislated breeds (53.3%). Finally, non-legislated breeds (92.9%) were more likely to bite when guarding an object compared to legislated breeds (7.1%).

**Table 1:** Sample sizes and incident percentages for dog breeds reported for dog bites in order of frequency for medium and large breeds^a^

Breed (Non-legislated)	Incidents	Breed (Legislated)	Incidents
	n(%)		n(%)
Border Collie	26(18.5)	German Shepherd	28(20)
Labrador Retriever	14(10)	Rottweiler	6(4.2)
Cocker Spaniel	5(3.5)	American Staffordshire Terrier	3(2.1)
Shetland Sheepdog	5(3.5)	Akita	2(1.4)
Boxer	4(2.8)	Doberman Pinscher	1(0.7)
English Springer Spaniel	4(2.8)		
Golden Retriever	4(2.8)		
Irish Red Setter	4(2.8)		
Poodle	4(2.8)		
Rough Collie	3(2.1)		
Scottish terrier	3(2.1)		
Beagle	2(1.4)		
Welsh Terrier	2(1.4)		
Bearded Collie	1(0.7)		
Black and Tan Hound	1(0.7)		
Bulldog	1(0.7)		
Chesapeake Bay Retriever	1(0.7)		
Clumber Spaniel	1(0.7)		
English Pointer	1(0.7)		
Foxhound	1(0.7)		
German Shorthaired Pointer	1(0.7)		
Greyhound	1(0.7)		
Irish Terrier	1(0.7)		
Leonberger	1(0.7)		
Old Danish Pointer	1(0.7)		
Old English Sheepdog	1(0.7)		
Pyrenean Mastiff	1(0.7)		
Shiba Inu	1(0.7)		
Siberian husky	1(0.7)		
Tibetan Terrier	1(0.7)		
Weimaraner	1(0.7)		
Wheaton terrier	1(0.7)		
Whippet	1(0.7)		

^a^Note: Breed risks cannot be inferred from this data as total populations are unknown

**Table 2:** Sample sizes and incident percentages for age categories, anatomical bite locations, and triggers for biting

Age (years)	Non-Legislated	Legislated	Bite location	Non-Legislated	Legislated	Trigger for bite	Non-Legislated	Legislated
	*n* (%)^a^	*n* (%)^a^		*n* (%)^a^	*n* (%)^a^		*n* (%)^a^	*n* (%)^a^
0–14	36(36)	20(50)	Hand/lower arm	40(40)	15(37.5)	Do not know	22(22)	14(35)
15–29	23(23)	9(22.5)	Lower leg/ft/ankle	28 (28)	6(15)	Dog was angry	8(8)	7(17.5)
30–44	22(22)	7(17.5)	Upper leg/torso	16(16)	10(25)	Dog was afraid	16(16)	1(2.5)
45–59	17(17)	4(10)	Neck/head/face	13(13)	5(12.5)	Dog was guarding its home	11(11)	6(15)
60–74	1(1)	0	Multiple locations	1(1)	4(10)	Dog was guarding an object	13(13)	1(2.5)
75–99	1(1)	0	Upper arm/shoulder	2(2)	0	Dog was fighting with another dog	8(8)	1(2.5)
						Dog was playing	8(8)	1(2.5)
						Dog was in pain	6(6)	1(2.5)
						Security dog carrying out duties	1(1)	3(7.5)
						Dog was chasing (predatory behaviour)	1(1)	0
						Multiple reasons	6(6)	2(5)
						Dog was instructed to attack	0	1(2.5)
						Dog was guarding puppies	0	2(5)

^a^Only valid responses are used for analyses, therefore totals may not add to total sample size (*N* = 140)

### Victim’s relationship with dog, geographical location and owner presence

No greater likelihood of group differences with respect to relationship with the dog was observed (*P* = 0.082). Examination of geographical location and owner presence revealed a significant effect (*P* = 0.036; see Table 3). Examination of residuals revealed that bites were more likely to occur when the owner was present on own property for non-legislated breeds (95%) than for legislated breeds (5%). In addition, non-legislated breeds (100%) were more likely to bite on a business premises (e.g. vets, groomers) compared to legislated breeds (0%; see Table 3).

**Table 3:** Sample sizes and incident percentages for victim’s relationship with dog, geographical location, and owner presence

Victims relationship with the dog	Non-Legislated	Legislated	Geographical location and owner presence	Non-legislated	Legislated
	n (%)^a^	n (%)^a^		n (%)^a^	n (%)^a^
Unfamiliar dog	36(37.1)	23(62.2)	Dog bit on public property, owner was absent	13(17.3)	6(23.1)
Familiar Dog	38(39.2)	9(24.3)	Dog bit on own property, owner was absent	12(16)	7(26.9)
Own dog (in possession more than 3 months)	18(18.6)	4(10.8)	Dog bit on public property, owner was present	10(13.3)	8(30.8)
Own dog (in possession less than 3 months)	5(5.2)	1(2.7)	Dog bit on own property, owner was present	19(25.3)	1(3.8)
			Dog bit owner	16(21.3)	4(15.4)
			Dog bit on dog business premises, professional present	5(6.7)	0

^a^Only valid responses are used for analyses, therefore totals may not add to total sample size (*N* = 140)

### Behavioural history and authority involvement

No significant difference in the likelihood of legislated or non-legislated having a history of aggression was observed (*P* = 0.349). A significant difference was observed between both legislated and non-legislated groups with respect to being reported to authorities before the bite (*P* = 0.009; see Table 4). Non-legislated breeds (79.5%) were more likely to not be reported to any authorities before biting compared to legislated breeds (20.5%). In addition, a significant difference in post bite reporting was observed (*P* = 0.032). Following the bite, non-legislated breeds (80%) were less likely to be reported to any authorities compared to legislated breeds (20%). No significant difference in known outcome for the dog was observed (*P* = 0.121). Legislated and non-legislated dogs were no more likely than one another to bite again (*P* = 0.238). In addition, no significantly greater likelihood of seeing a professional trainer or behaviourist was observed between both groups (*P* = 0.579).

**Table 4:** Sample sizes and incident percentages for behavioural history and authority involvement

History of aggression	Non-Legislated	Legislated	Reported before bite	Non-legislated	Legislated	Reported after bite	Non-legislated	Legislated
	n (%)^a^	n (%)^a^		n (%)^a^	n (%)^a^		n(%^)a^	n(%^)a^
No history of aggression	28(28)	6(15)	Not reported before bite	58(58)	15(37.5)	Not reported after bite	72(72.7)	18(45)
Yes, had behaved aggressively	20(20)	10(25)	Do not know	40(40)	20(50)	Do not know	20(20.2)	15(37.5)
Yes, had bitten	15(15)	4(10)	Yes, reported to police	2(2)	3(7.5)	Yes, reported to police	4(4)	5(12.5)
Do not know	37(37)	20(50)	Yes, reported to animal control	0	2(5)	Yes, reported to animal control	3(3)	2(5)

^a^Only valid responses are used for analyses, therefore totals may not add to total sample size (*N* = 140)

### Type of bite and medical treatment required

Regarding type of bite, neither legislated breeds nor non-legislated breeds were more likely than the other to inflict a differing bite type with greater severity (*P* = 0.604; see Table 5). In addition, neither legislated breeds nor non-legislated breeds were more likely to inflict a bite requiring greater medical attention than the other (*P* = 0.122; see Table 5).

**Table 5:** Sample sizes and incident percentages for type of bite and medical treatment required

Type of bite	Non-Legislated	Legislated	Medical treatment required	Non-legislated	Legislated
	n (%)^a^	n (%)^a^		n (%)^a^	n (%)^a^
Level 2	23(23)	9(22.5)	No treatment/at home treatment	47(47)	12(30)
Level 3	47(47)	22(55)	GP visit/antibiotics/tetanus shot	28(28)	17(42.5)
Level 4	25(25)	6(15)	Stitches/staples/glue/regular wound dressing	21(21)	11(27.5)
Level 5	5(5)	3(7.5)	Serious medical treatment/surgery/fractures/repeat hospital visits	4(4)	0

^a^Only valid responses are used for analyses, therefore totals may not add to total sample size (*N* = 140)

### Dog control officer survey

With the exception of one officer’s shelter who reported not recording information pertaining to breeds, dog breed identification was reported to be conducted through subjective measures, including visual identification (see Table 6). Over half (59%) of the dog control officers felt that breed-specific legislation is effective. Similarly, over half (56%) reported that they believed legislated breeds had the capability to inflict greater injuries and more severe damage if biting compared to non-legislated breeds of similar size. In addition, 19% of officers surveyed felt legislated breeds were more aggressive than non-legislated breeds. With respect to accepting surrenders, one officer reported that their shelter did not accept surrenders of legislated dog breeds from the public. A further officer indicated that the shelter they operate only allow the rehoming of certain legislated breeds.

**Table 6:** Sample sizes and incident percentages for dog control officer survey

	*n* (%)^a^
How is a dog’s breed identified?	
Officer visually identifies the breeds	5(29)
Officer visually identifies the breeds and asks owner	6(35)
Officer visually identifies, asks owner and checks records	5(29)
Do not record breed	1(6)
Do you currently accept surrenders of legislated dog breeds from the public?	
Yes	15(94)
No	1(6)
Missing	1
Do you allow the rehoming of legislated dog breeds?	
Yes	15(94)
No (some breeds)	1(6)
Missing	1
Do you believe breed specific legislation is effective in reducing dog bites in Ireland	
Yes	10(59)
No	7(41)
In your experience, do you believe legislated dog breeds can inflict greater injuries or physical damage compared to non-legislated breeds of similar size?	
Yes	
No	9(56)
Missing	7(44)1
In your experience, are legislated dog breeds more aggressive than non-legislated breeds?	
Yes	3(19)
No	13(81)
Missing	1

## Discussion

Present findings suggest no difference between biting legislated and non-legislated dog breeds for; age when the victim was bitten, bite location, relationship with the dog, history of aggression, known outcome for the dog, if the dog bit again, and seeing a professional trainer or behaviourist. Both Border Collies and German Shepherds are observed as being involved in a greater number of bite incidents within this sample. Given complete dog breed populations are unknown for all breeds listed, risk relating to frequency of bites from any breeds within this study cannot be computed. Indeed, in line with research examining dog bite populations, the breeds reported for biting are comparatively in line with the more popular dog breeds within a population in Ireland [7]. Regarding breed grouping comparisons, legislated breeds were perceived as biting due to being more aggressive and less fearful than non-legislated breeds. In addition, non-legislated breeds were more likely to be reported as being triggered to bite due to guarding an object. While dogs similarly signal their intent to bite [28], there was a significant difference observed between groups. The impact of public perceptions and stereotypes of risk relating to dog breeds cannot be understated [29]. While providing an agenda for future work, the observed effect may be due to the perceptions of breed risk rather than exhibited behaviour. Legislated breeds could well be perceived as aggressive and less fearful given their reinforced stereotype. While speculative, legislated breed owners may be more likely to address unwanted guarding behaviour compared to non-legislated breed owners.

Examination of geographical location and owner presence revealed significant findings. Non-legislated breeds were observed as more likely to inflict a bite on a business premises compared to legislated breeds. Bites were also more likely to occur with the owner present on own property for non-legislated breeds compared to legislated breeds. Intriguingly, this may suggest differing perceptions of responsibility for owners of dogs from both groups. Owners from legislated breeds may be more likely to take precautions (e.g. continuous supervision) with their dogs compared to non-legislated owners. Individuals may thus perceive non-legislated breeds as safer and having a greater tolerance, which is reinforced by their non-legislated status. This is supported by authority involvement findings where non-legislated breeds were significantly less likely to be reported to any authorities both before, and after the bite occurred. This suggests a significantly lesser risk is associated with non-legislated breeds, thus potentially reinforcing the authorities’ perception of risk relating to these breeds.

No significant difference was observed between legislated and non-legislated dog breeds for the medical attention required following a bite. In addition, no significant difference was observed between legislated and non-legislated breeds for the type of bite inflicted. In other words, legislated breeds were found not to have a greater likelihood of inflicting greater injury and a differing bite type compared to non-legislated breeds. While a greater ability to inflict bites of greater severity and requiring more medical attention is frequently attributed to legislated breeds, these results do not provide evidence in support of these assertions.

Regarding dog control, one officer reported not recording dog-breed information. The remaining officers relied upon subjective measures, including visual identification methods of determining breeds. A landmark study conducted by Scott and Fuller [30], found that the offspring of two different purebred dogs frequently bear no resemblance whatsoever to either breed. As such, it is unclear how officers would be capable of accurately identifying mixed breed dogs. Over half of the officers surveyed reported feeling that current breed-specific legislation is effective in reducing dog bites. Over half of officers also believed that legislated breeds have an ability to inflict greater injuries compared to non-legislated breeds of similar size. Less than a quarter of officers felt legislated breeds were in fact more aggressive than non-legislated breeds. While a number of officers did not complete the survey; one officer reported that their shelter does not accept surrenders of legislated breeds from the public, with a further not allowing the rehoming of legislated dog breeds.

It is important to not understate the potential knock-on effect targeting dog breeds may have. Assumptions about the supposed ‘aggressive’ or ‘able to inflict greater injury’ nature of legislated breeds or the ‘less capable of inflicting significant injury’ or ‘docile’ temperament of non-legislated breeds, may be associated with differing interactions across breeds. Consider a scenario where a dog begins to bark following encroachment on its personal space by a human. If the dog is a legislated breed, the individual may perceive such behaviour as symptomatic of the ‘aggressive nature’ of such breeds. On the other hand, the individual may fail to recognise such warning signals from a non-legislated breed. In both instances, the individual may not recognise the trigger, interpret the dog’s behaviour correctly, or respond appropriately, thereby increasing the risk of this interaction resulting in the dog biting. In other words, the criterion for interacting with a dog may be incorrectly rule governed by its breed rather than actual exhibited behaviour, which in turn is being reinforced by the breed-specific legislation.

Potential limitations which also provide an agenda for future work must be duly noted. Methodologies which employ any retrospective self-report measures can be particularly open to threats from recall bias. Indeed, while every attempt to minimise such threats to memory recall by incorporating detailed and specified survey questions [31], potential threats due to recall may have occurred. Additionally, while only reported pure-bred dogs were assessed, the potential threat of breed misidentification cannot be ruled out. Indeed, further effects may also emerge with an increase in sample size. While the present study was adequately powered to detect medium to large effects, further significant small effect sizes may emerge with an increased sample. Indeed, a further consideration is the implications and relevance of examining small dog breeds in future research. Much research has indicated that small dog breeds are frequently identified as displaying higher levels of aggression compared to larger dog breeds [32]. Indeed, human fatalities have also been inflicted by small breed dogs, and as such future research examining associations with smaller dog breeds would be of importance. Further research is also warranted in addressing knowledge of determining the emotional responsivity of dogs. Research would be required to replicate present findings, and potentially provide further analyses relating to the differing contexts which may have characterised the expressed aggression.

## Conclusions

The present study provides evidence that the targeting of dog breeds as a dog bite mitigation strategy may pose significant negative consequences relating to perceptions of risk and reporting behaviour. Its introduction in Ireland poses further wide reaching negative consequences; animal welfare concerns relating to dog pounds not rehoming and accepting surrenders of these breeds (see Table 6), restrictions affecting disability/assistance dogs, and owner housing restrictions [33] among others. A legislative dog-bite mitigation strategy whose purpose is to provide safeguards to the public through a reporting system, should avoid putting divisive mechanisms across responsible dog-owner populations. Doing so will make the identification of dogs likely to bite difficult and as observed within this study, will lead to a distinct bias in dog bites reported to authorities. The increased perception of threat from specific breeds, and the lack of perceived threat from other breeds are essentially two sides to the same counterproductive coin. The increasing trend in dog-bite hospitalisations in Ireland is alarming [1], yet unsurprising. Evidence based breed-neutral alternatives exist, which target multi-factorial risk factors, and as such should be enacted [34–37]. It is recommended a public policy mechanism which categorises potentially dangerous dogs based on their exhibited behaviour is enacted [38].
