# Erratum to: Dog bite injuries to humans and the use of breed-specific legislation: a comparison of bites from legislated and non-legislated dog breeds

**DOI:** 10.1186/s13620-017-0104-y

**Published:** 2017-08-15

**Authors:** Nanci Creedon, Páraic S. Ó Súilleabháin

**Affiliations:** 1 Department of Dog Behaviour, Creedons College, Vicars Road, Cork City, Ireland; 2 School of Psychology, National University of Ireland, Galway, University Road, Galway, Ireland

## Erratum

After publication of the original article [1] it was brought to our attention that the following errors had occurred:

1. Author Páraic S. Ó Súilleabháin was incorrectly included as Páraic S. Ó’Súilleabháin. The correct spelling of the surname is included in the author list of this erratum and updated in the original article.
2. Furthermore, number 1 was omitted from Table 6, question “In your experience, do you believe legislated dog breeds can inflict greater injuries or physical damage compared to non-legislated breeds of similar size?”, response “yes”. The corrected table is provided here and has been updated in the original article.

**Table 6:** Sample sizes and incident percentages for dog control officer survey

	n (%)^a^
How is a dog’s breed identified?	
Officer visually identifies the breeds	5(29)
Officer visually identifies the breeds and asks owner	6(35)
Officer visually identifies, asks owner and checks records	5(29)
Do not record breed	1(6)
Do you currently accept surrenders of legislated dog breeds from the public?	
Yes	15(94)
No	1(6)
Missing	1
Do you allow the rehoming of legislated dog breeds?	
Yes	15(94)
No (some breeds)	1(6)
Missing	1
Do you believe breed specific legislation is effective in reducing dog bites in Ireland	
Yes	10(59)
No	7(41)
In your experience, do you believe legislated dog breeds can inflict greater injuries or physical damage compared to non-legislated breeds of similar size?	
Yes	1
No	9(56)
Missing	7(44)1
In your experience, are legislated dog breeds more aggressive than non-legislated breeds?	
Yes	3(19)
No	13(81)
Missing	1
